# Complete Transthoracic Echocardiography for the Assessment and Guidance of Percutaneous Atrial Septal Defect Closure in Adults without Balloon Sizing: An Observed Study with a 10-Year Follow-Up

**DOI:** 10.3390/jcdd10080321

**Published:** 2023-07-28

**Authors:** Lin-Feng Xie, Yong Lin, Mei-Fang Chen, Gui-Can Zhang

**Affiliations:** 1 Department of Cardiovascular Surgery, Fujian Medical University Union Hospital, Fuzhou 350000, China; fjmuxlf@163.com (L.-F.X.); birdman1983@163.com (Y.L.); 13799400450@163.com (M.-F.C.); 2 Key Laboratory of Cardio-Thoracic Surgery (Fujian Medical University), Fujian Province University, Fuzhou 350000, China; 3 Fujian Provincial Center for Cardiovascular Medicine, Fuzhou 350000, China

**Keywords:** heart septal defects, atrial, cardiac catheterization, echocardiography, balloon occlusion, outcomes

## Abstract

Objectives: This study aims to determine if complete transthoracic echocardiography (TTE)-guided percutaneous atrial septal defect (ASD) closure without balloon sizing could be safe and efficacious in adult patients. Methods: A total of 551 patients with ASDs were enrolled in this study, of which 438 patients underwent percutaneous ASD closure. Patients who received TTE-guided percutaneous ASD closure were classified into group T, and those who underwent a procedure that was guided by transesophageal echocardiography (TEE) were classified into group E. The clinical characteristics and the outcomes of the patients were analyzed. Results: The characteristics were comparable at baseline, except for the body mass index (BMI) (21.6 ± 5.3 vs. 23.8 ± 7.1, *p* < 0.001) between group T and group E. No significant difference was observed between the two groups regarding in-hospital outcomes, except for the duration of the procedure (29.8 ± 15.3 min vs. 41.5 ± 20.4 min), the length of stay in the hospital (2.1 ± 2.3 d vs. 2.9 ± 2.6 d), and hospital costs (USD 6233.3 ± 312.4 vs. USD 6673.7 ± 446.9). There were no significant differences in the incidences of long-term complications, cardiac chamber sizes, and tricuspid regurgitation severity between the patients in the two groups during the 10-year follow-up period. Conclusion: TTE may be as safe and efficacious as TEE for the assessment and guidance of percutaneous ASD closure without balloon sizing in adult patients with lower BMIs who are commonly found in East Asia.

## 1. Introduction

Atrial septal defects (ASDs) account for 25% to 30% of congenital heart defects in the adult population [1]. In 1976, King’s group [2] reported the first successful percutaneous ASD closure (PAC). Currently, PAC has become a prevalent intervention therapy for ASDs [3]. Balloon sizing and transesophageal echocardiography (TEE) remain the gold standards of peri-procedural assessments and real-time guidance. In order to avoid X-ray exposure, general anesthesia, and TEE-related complications, transthoracic echocardiography (TTE) has been applied during ASD closure for several years [4,5]. However, due to unsatisfactory imaging, TTE-guided PAC is controversial and is frequently applied in children [5,6]. Nevertheless, complete TTE-guided PAC without fluoroscopy and balloon sizing has been successfully performed at our center for more than 10 years [7,8]. Because our previous report lacked control groups and long-term follow-up, the aim of this study was to determine if complete TTE-guided PAC without balloon sizing could be safe and efficacious in an adult population.

## 2. Materials and Methods

### 2.1. Patients

**Figure 1:**
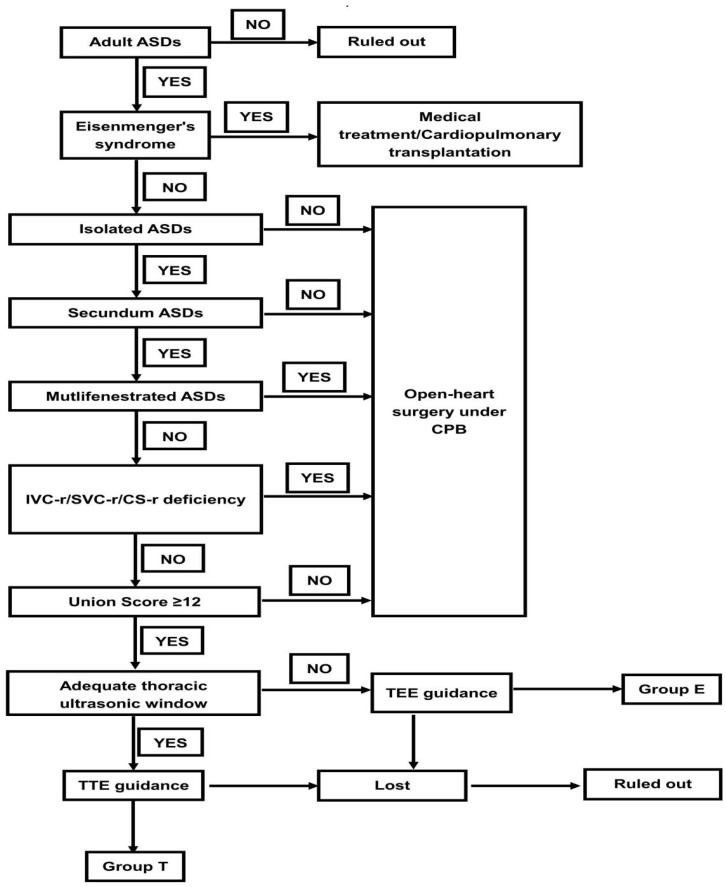
Flow chart. The diagram of patient selection is described in the figure. Referring to a quantitative procedural indication for percutaneous transcatheter ASD closure, which is described in Table 1. ASDs: atrial septal defects; TTE: transthoracic echocardiography; PTC: percutaneous transcatheter closure; TEE: transesophageal echocardiography.

Between 1 January 2007 and 31 December 2011, a total of 551 consecutive patients with ASDs in southeast China were enrolled in this study. All patients were diagnosed with isolated secundum ASDs, and echocardiography examination showed evidence of right ventricular-volume overloading due to a significant left-to-right shunt. We excluded any patients with deficient inferior vena cava (IVC) rims, deficient superior vena cava (SVC) rims, deficient coronary sinus (CS) rims or other congenital heart diseases, Eisenmenger’s syndrome, and those who had primum or multiple ASDs. Patients who were younger than 18 years and those who were lost during the period of the follow-up were also excluded from this study. Patients who received TTE-guided PAC were classified into group T. Patients who did not have adequate TTE windows were scheduled for TEE-guided procedures, and they were classified into group E (Figure 1). The baseline demographic characteristics, the morphology of the defects, cardiac parameters, and the outcomes of the patients were analyzed. 

This study was approved by the ethics committee of the Union Hospital of Fujian Medical University (Fuzhou, China) and conformed to the Declaration of Helsinki, and this retrospective review of patient data did not require written informed consent from the participants, which is in accordance with national guidelines.

### 2.2. Definition

Procedural success was defined as the presence of the following criteria: successful delivery of the occlusion device without peri-procedural complications and well-positioned device confirmed via TTE in the first 24 h after surgery. A deficient rim was defined as <5 mm in length. In-hospital outcomes included procedural times, device size, the success rate at the first attempt, device size change, conversion to open-chest surgery, transfusion, shunt inside the device, device embolization, hemolysis, device-related valve regurgitation, residual shunt, atrial arrhythmias, third-degree atrioventricular block requiring pacemaker implantation, cardiac rupture, heart failure, and death. The procedural time was defined as the duration starting from the patient’s entry into the operating room to their exit from the operating room. Long-term outcomes included device erosions, residual shunting, thrombosis events, heart failure, atrial arrhythmias, third-degree atrioventricular block, stroke, valve regurgitation, and death. All events were new during the onset or had obviously deteriorated compared with the pre-procedural status.

### 2.3. Pre-Procedural Assessment

**Figure 2:**
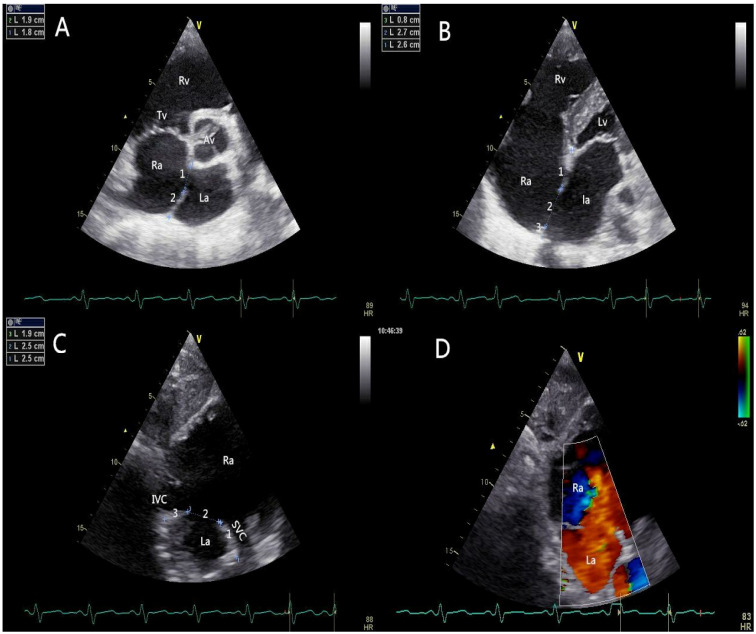
Pre-procedural transthoracic echocardiographic assessment. (**A**) The short-axis left parasternal view of aortic valve delineated the aortic rim and the posterior rim of the defect; (**B**) The four-chamber view showed the atrioventricular valve rim and the upper rim; (**C**) The short axis subcostal view showed the SVC rim and IVC rim; (**D**) The short axis subcostal view (coloured) showed the blood flow cross the atrial septum (left to right). AV: aortic valve; RA: right atrium; LA: left atrium; RV: right ventricle; LV: left ventricle; TV: tricuspid valve; IVC: inferior vena cava; SVC: superior vena cava.

All patients should be evaluated using TTE in terms of defect size, location, rims, adjacent structures, and cardiac function. The edge of the defect was divided into six areas according to their locations or neighboring structures (Figure 2): upper rim (Up-r), atrioventricular valve rim (AVV-r), aortic rim (Ao-r), posterior rim (Po-r), SVC rim (SVC-r), and IVC rim (IVC-r). A floppy septum was considered a normal rim. A “Union Score” was utilized to select patients who were suitable for PAC (Table 1).

The figure shows the transthoracic echocardiographic view of ASD without an aortic rim in a 73-year-old patient (Union Score = 0 + 3 + 3 + 2 + 3 + 3 = 14 points). The short-axis left parasternal view of the AV delineated the entire length of the ASD (3A-1), the aortic rim (deficient, 0 points), and the posterior rim (3A-2, 19 mm, 3 points). The four-chamber view shows the atrioventricular valve rim (3B-3, 26 mm, 3 points) and the upper rim (3B-1, 8 mm, 2 points), and the maximum length of the ASD (3B-2) could be observed in this view. The short-axis subcostal view shows the length of the defect (3C-2), the SVC rim (19 mm, 3 points), and the IVC rim (25 mm, 3 points). The figure shows the transthoracic echocardiographic view of the ASD with an adequate SVC rim and an adequate IVC rim. The short-axis subcostal view (colored) shows the blood flow across the atrial septum (left to right) (3D).

In addition, the anomalous pulmonary venous connection should be excluded, especially for ASDs with a short IVC-r. The severity of pre-procedural valve regurgitation was assessed using color flow Doppler. Prominent eustachian valve tissue on the right side of the atrial septum that would interfere with the catheter’s position and device placement was noted and communicated to the cardiac interventionalists.

### 2.4. Catheterization Therapy Process

**Figure 3:**
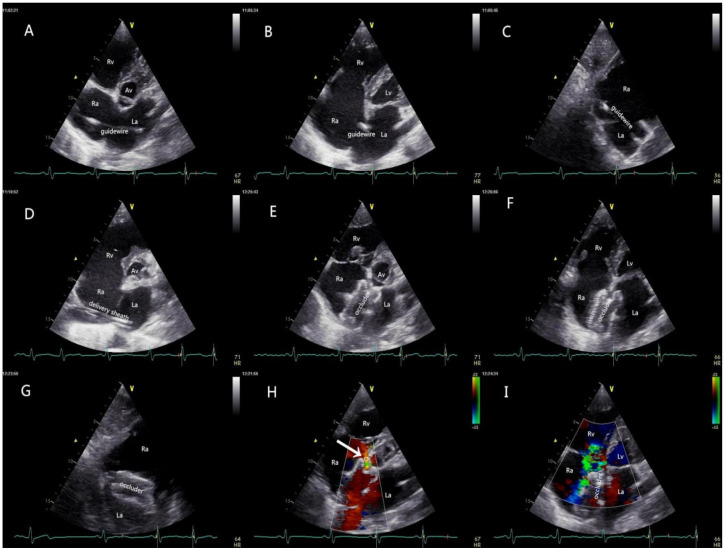
Procedural protocol. (**A**) The guidewire was inserted from the RA into the LA via the femoral vein under the guidance of TTE; (**B**,**C**) the tail end of the high-echo guidewire was confirmed to be inside the LA in the four-chamber view and the short-axis subcostal view; (**D**) a delivery sheath was inserted along the guide-wire into the LA, and then the inner core and the guidewire were withdrawn; (**E**) the occluder was delivered carefully through the sheath under the real-time guidance of TTE, and the LA disc was opened; (**F**,**G**) the occluder was completely released, and the two discs of the occluder were accurately parallel to the plane of the atrium septal; (**H**) the blood flow from CS was fluent (white arrow); (**I**) the shunt between RA and LA disappeared, and no mitral regurgitation was detected with TTE. Mild tricuspid regurgitation still existed, as confirmed before the interventional catheterization. TTE: transthoracic echocardiography; RA: right atrium; LA: left atrium; RV: right ventricle; LV: left ventricle; TV: tricuspid valve; AV: aortic valve; CS: coronary sinus.

Patients with an adequate thoracic ultrasonic window received local anesthesia and underwent TTE-guided PAC, and the remainder received the procedure under traditional TEE guidance and general anesthesia. Right heart and peripheral artery catheterization were routinely performed to achieve perioperative hemodynamic monitoring. The patient was then placed in a 30-degree right-chest elevated position. The size of the Amplatzer Septal Occluder (LZASD, SHANGDONG VISEE MEDICAL DEVICES Co., Ltd., China) was completely based on the result of echocardiography (oversized by 4 mm to 6 mm more than the maximum dimension of the defect) rather than balloon sizing in all patients. The procedure is demonstrated in Figure 3. The patients receiving regional anesthesia were sent directly to the general ward, and those who underwent general anesthesia were sent to the ICU. Anticoagulation via the oral administration of dipyridamole or aspirin was carried out for 3 months after the procedure.

### 2.5. Follow-Up

TTE and electrocardiography were performed on the first day after surgery. Telephone contact or outpatient review regarding the living status and TTE and electrocardiography examinations were maintained every 3 months in the 1st year and every 6 months after the 2nd year after discharge. The follow-up period lasted for at least 10 years. 

### 2.6. Statistical Analysis

Statistical analyses were performed using SPSS 25.0 for Mac (Chicago, IL, USA) and GraphPad Prism 8.0.1 (San Diego, CA, USA). Continuous variables were presented as mean ± SD, and the categorical variables were presented as numbers and percentages. Descriptive statistical analyses, as well as *t*-tests, were used to analyze the measurement data. The chi-square test or Fisher’s exact test was used to analyze numerical data. One-way repeated measures ANOVA was used to compare the difference between the patients in two groups in the echocardiographic parameters during the follow-up period. Kaplan–Meier curves were used to plot the freedom from long-term complications. A two-tailed *p* < 0.05 was considered statistically significant.

## 3. Results

### 3.1. Baseline Demographic Characteristics 

In total, 113 patients were excluded from this study: 39 cases of primum ASDs, 5 cases of patients being younger than 18 years old; 26 cases of ASDs with deficient IVC/SVC/CS rims; 4 cases of anomalous pulmonary venous drainage; 2 cases of serious mitral valve lesions requiring surgical repair; 1 case of coronary heart disease requiring coronary artery bypass grafting; 5 cases of Eisenmenger’s syndrome; 29 cases with a Union Score of lower than 12 points; and 2 cases who were lost after discharge. Four hundred and thirty-eight patients were divided into two groups. Three hundred and thirteen patients who received TTE-guided PAC were classified into group T, and one hundred and twenty-five patients who underwent the procedure under TEE guidance were classified into group E. There were no significant differences between the patients in the two groups in terms of clinical characteristics except for the body mass index (BMI) (Table 2).

### 3.2. In-Hospital Outcomes

No significant difference was found between the two groups regarding in-hospital outcomes except for the procedural time, the length of stay in the hospital, and hospital costs. In group T, two patients (2/313, 0.6%) converted to open-chest surgery because of shedding of the occlusion device and a right atrium rupture. A residual shunt was observed in one case (1/313, 0.3%), in which they received a larger device in the operating room as soon as they were diagnosed; then, the shunt disappeared. One patient (1/313, 0.3%) was confirmed to exhibit mild hemolysis within 24 h after the procedure and then received surgical repair under cardiopulmonary bypass. Two patients (2/313, 0.6%) experienced new-onset atrial arrhythmias within 3 days after the procedure. In group E, one case (1/125, 0.8%) had occluder shedding, one case (1/125, 0.8%) had third-degree atrioventricular block, one case (1/125, 0.8%) had a cardiac rupture, and all underwent surgical repair (3/125, 2.4%). One patient (1/125, 0.8%) experienced mild device-related tricuspid valve regurgitation during the procedure. One case (1/125, 0.8%) of new-onset atrial arrhythmia was observed 3 days after the procedure. One case (1/125, 0.8%) of heart failure occurred, and they received medical treatment and recovered 3 days later. The rest of the patients from the two groups underwent transcatheter closure successfully without any complications (Table 3).

### 3.3. Long-Term Outcomes

**Figure 4:**
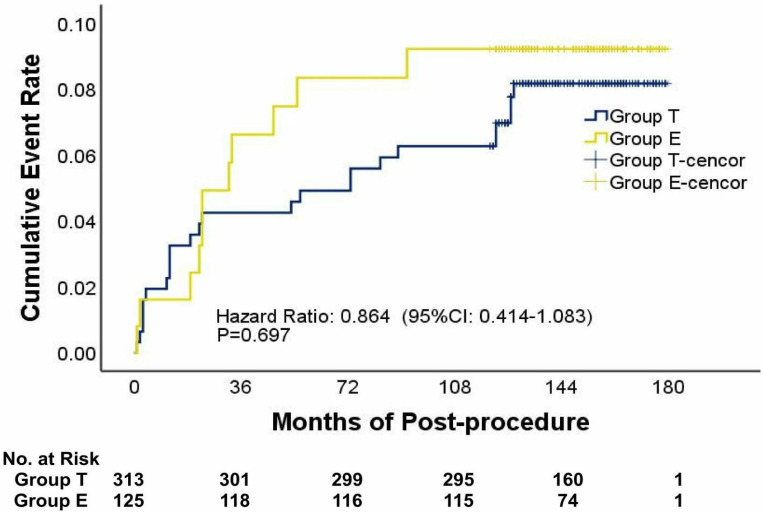
Cumulative event rate. Long-term outcomes and post-procedural adverse events included device erosions, residual shunting, thrombosis events, heart failure, atrial arrhythmias, third-degree atrial ventricular block, stroke, valve regurgitation, and death. All events were new during onset or had obviously deteriorated compared with the pre-procedural status.

**Figure 5:**
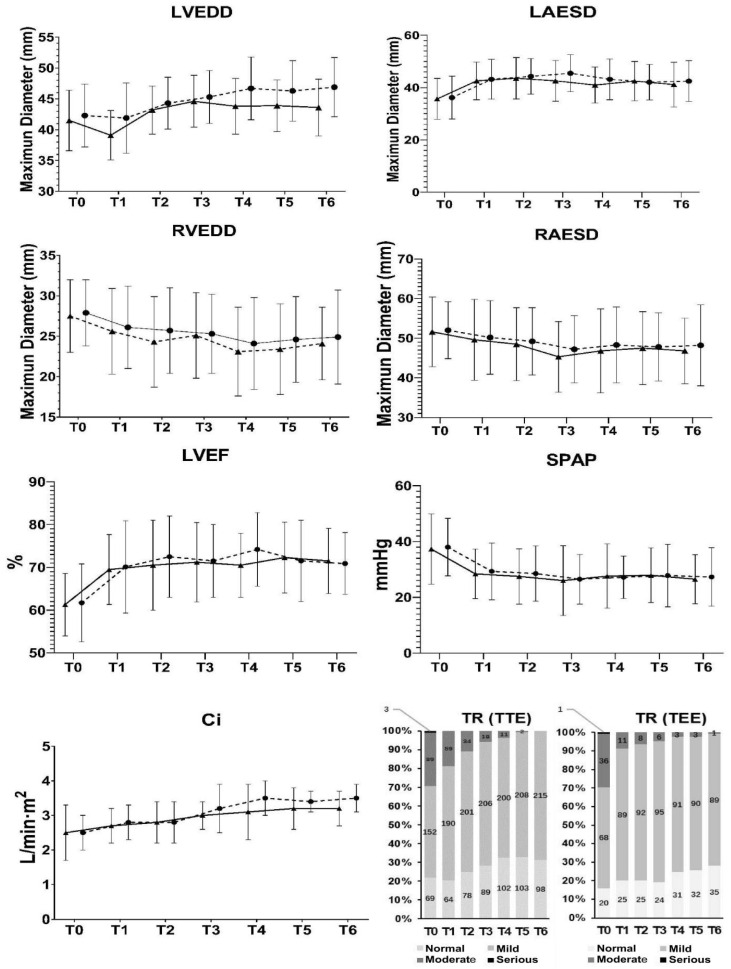
Changes in heart size and tricuspid function. TTE examinations were maintained every 3 months in the 1st year and every 6 months since the 2nd year after discharge. LEVDD: F = 1.176, *p* = 0.849; LAESD: F = 1.276, *p* = 0.775; RVEDD: 1.455, *p* = 0.660; RAESD: 1.608, *p* = 0.579; LVEF: 1.159, *p* = 0.863; SPAP: 1.060, *p* = 0.946; Ci: 2.211, *p* = 0.357. ▲: Group T; ●: group E; T0: pre-procedure; T1: immediate post-procedure; T2: 3 months after procedure; T3: 6 months after procedure; T4: 1 year after procedure; T5: 5 years after procedure; T6: 10 years after procedure. LVEDD: End-diastolic diameter of left ventricle; LAESD: end-systolic diameter of left atrium; RVEDD: end-diastolic diameter of right ventricle; RAESD: end-systolic diameter of right atrium; LVEF: left ventricular ejection fraction; SPAP: systolic pulmonary artery pressure; Ci: cardiac index; TR: tricuspid regurgitation; TTE: transthoracic echocardiography; TEE: transesophageal echocardiography.

There were no significant differences between the two groups in terms of long-term outcomes. A total of 38 cases (38/438, 8.7%) of long-term complications were discovered as follows: 2 cases of device erosions (2/438, 0.5%), 2 cases of thrombosis events (2/438, 0.5%), 3 cases of heart failure (3/438, 0.7%), 22 cases of atrial arrhythmias (22/438, 5.0%), 5 cases of third-degree atrioventricular block (5/438, 1.1%), 1 case of stroke (1/438, 0.2%), and 3 cases of valve regurgitation (3/438, 0.7%) (Table 3). Kaplan–Meier plots also revealed that there was no significant difference between the patients in group T and group E during the 10-year follow-up period with respect to the proportion of patients with long-term complications (log-rank *χ*^2^ = 0.210, *p* = 0.647) (Figure 4). One-way repeated measures ANOVA analysis revealed that there was no significant difference between the patients in the two groups with respect to the changes in cardiac chamber sizes and tricuspid regurgitation severity before and after the procedure in this study (Figure 5).

## 4. Discussion

Percutaneous transcatheter closure has become prevalent in interventional treatment for isolated secundum ASDs, which is performed under both TEE guidance and balloon sizing in most medical centers [9,10]. However, TEE requires general anesthesia, which is related to laryngospasm, aspiration pneumonia, and esophageal trauma [11], and balloon sizing leads to excessive X-ray exposure. Intracardiac echocardiography is another available guiding technology that does not use fluoroscopy, and it can provide a clear view of cardiac structures. Nevertheless, this technique is expensive and requires the placement of a second large venous access [12]. Three-dimensional printing was recently reported as an aid in transcatheter ASD closure, especially for defects with non-integrated rims [13]. However, it is very difficult to produce a three-dimensional model of mitral valve leaflets because of the limitation of the spatial and temporal resolution of multislice computed tomography. The encroachment of the mitral valve by the closure device has become a potential risk of this technology. 

TTE is non-invasive and does not require general anesthesia in most cases, and it was not surprising that the hospital costs in group T were lower than those in group E. Bateau AE et al. [14] verified that compared with TEE, the transcatheter closure of ASD could also be performed under TTE guidance in experienced centers, even in patients with large defect sizes. Li et al. [15] reported that compared with historical controls under TEE guidance, the overall complication incidence did not differ with TTE guidance, although the absence of a sufficient superior-anterior rim in patients undergoing PAC using fluoroscopic and TTE guidance is associated with slightly and non-statistically greater device malposition and migration. In addition, it is worth noting that TTE has an advantage in the observation of IVC and its nearby structures, which facilitates the identification of IVC-type ASDs or the confirmation of the guidewire’s entrance through the IVC orifice. Additionally, the significant difference in BMI between the two groups revealed that the transthoracic windows were adequate for guides, especially in lower BMI patients who are frequently encountered in East Asia.

The most important decision-making step in PAC is the precise sizing of the defect and the choice of an appropriate device [16]. The balloon sizing of defects was an established method in the protocol. However, it may cause an enlargement of the defect by tearing the surrounding rim tissues and causing balloon rupture, cerebral microembolism, and cardiac perforation [16,17,18,19,20,21]. Additionally, balloon sizing increases the duration of the procedure and the exposure time of fluoroscopy. Furthermore, the measurement may not be accurate because the waist of the balloon may not have a good profile with respect to the cine projection, especially in patients with large ASDs [22,23]. Moreover, bradycardia and hypotension may occur during the prolonged inflation of the balloon due to the obstruction in the diastolic filling [24]. Recently, many cardiac interventionists have performed PAC successfully under echocardiographic guidance compared with the balloon sizing method. Jia et al. [25] demonstrated that complete TTE-guided PAC could be an easy choice for some simple ASD patients. Wang et al. [26] reported a series of patients whose ASDs were closed without balloon sizing. They concluded that there was no difference in the success or complication rate between those with or without balloon sizing and suggested that balloon sizing was not necessary. Jeong Yoon Jang et al. [27] perceived that transcatheter ASD closure using the three-dimensional TEE-derived formula without balloon sizing is clinically feasible and safe. However, caution should be taken to exclude unfavorable features of the defects, including tunnel-shaped ASDs resembling the shape of patent foramen ovale, the interatrial septum showing aneurysms, and elliptical ASDs located along the margin of the aneurysm. Continued pressure or friction from improper occluder devices may cause edema, inflammation, or scarring of the atrioventricular node, leading to third-degree atrioventricular block, which is not allowed in our center. Therefore, five cases with this serious complication underwent surgical repairs of the ASD immediately. In addition, there were no differences between the patients in the two groups with regard to the incidence of third-degree atrioventricular block. Hence, we believe that complete TTE in the assessment of ASD size without balloon sizing is precise and instructive.

Secundum ASDs with non-integrated rims are not rare, and the appropriate candidates for PAC are still controversial. ASDs without IVC-r are regarded as a contraindication or indicate a high risk of conversion to open-heart surgery [28]. Pascal Amedro and his colleagues reported that only 44.4% of patients with non-integrated inferior-posterior rims had successful ASD closures, and 33.3% of patients presented major complications, including device embolization, pericardial effusion, and complete atrioventricular block. Therefore, ASDs with non-integrated inferior-posterior rims were not recommended for transcatheter closure by these authors [29]. However, a short IVC-r length is not always considered to be a procedural contraindication for transcatheter ASD closure if the other rims around the ASD can compensate for the anatomical insufficiency. Yasufumi Kijima et al. [9] performed PAC on 48 patients with a non-integrated IVC-r, and transcatheter closure was successfully accomplished in 43 patients (89.6%). In addition, it is worth mentioning that nearly two-thirds of patients who were enrolled in Yasufumi Kijima’s study also had Ao-r deficiency, but no significant difference in the occurrence of cardiovascular events was observed among patients with integrated rims and those with deficient Ao-r. Marco Pappa et al. [10] held the same view as Yasufumi Kijima, and they demonstrated that it was feasible and safe to proceed with transcatheter closure for ASDs with a deficient Po-r or Ao-r. Hence, ASDs with a non-integrated Ao-r are considered to be an indication of PAC [30]. In our study, the Union Score was applied for the selection of the patients, including those with deficient rims. Good results showed a wide range of applications in patient selection for PAC. 

There were several limitations in this study. First, this study is limited by its retrospective design, as it only involved a single center, and many confounders, such as demographic and anthropomorphic characteristics, were not controlled. A randomized multicenter study using a larger and more diverse population with ASDs is needed. Secondly, only Amplatzer septal occluders were used, and our results may not be simply extrapolated to other devices for PAC. Finally, compared with TEE, TTE does not perform as well in recognizing complications such as residual shunts or device malposition. Further studies will be needed to verify the clinical value of applying our method to other devices. 

## 5. Conclusions

In adult patients with lower BMIs who are commonly found in East Asia, the assessment and guidance of TTE may be as safe and efficacious as TEE in balloon-free PAC. The application of TTE appears to have economic and medical security advantages because of the avoidance of general anesthesia and the freedom from fluoroscopy exposure.

## Figures and Tables

**Table 1:** Quantitative indication for percutaneous ASD closure (Union Score).

	Length of the Rims and the Corresponding Values			
	0 mm	1–4 mm	5–9 mm	≥10 mm
**Parasternal short-axis view**				
Aortic rim	0	1	2	3
Posterior rim	0	1	2	3
**Apical four-chamber view**				
Atrioventricular valve rim	0	1	2	3
Upper rim	0	1	2	3
**Subcostal short-axis view**				
Inferior vena cava rim	0	1	2	3
Superior vena cava rim	0	1	2	3

The indication of percutaneous ASD closure was Union Score ≥ 12, and the contraindications were indicated by Union Score < 12 or if any of the inferior vena cava rim, superior vena cava rim, or coronary sinus rim were shorter than 5 mm. ASD: atrial septal defect.

**Table 2:** Clinical characteristics.

Category	Group T (*n* = 313)	Group E (*n* = 125)	*p*-Value
**Age, y**	39.3 ± 19.3	40.5 ± 19.6	0.559
**Male, *n* (%)**	105 (33.5)	39 (31.2)	0.654
**BMI**	21.6 ± 5.3	23.8 ± 7.1	<0.001
**Hypertension, *n* (%)**	36 (11.5)	15 (12.0)	>0.999
**Diabetes mellitus, *n* (%)**	11 (3.5)	6 (4.8)	0.585
**Coronary heart disease, *n* (%)**	5 (1.6)	3 (2.4)	0.694
**Cerebrovascular accident, *n* (%)**	3 (1.0)	1 (0.8)	>0.999
**Atrial arrhythmias, *n* (%)**	32 (10.2%)	18 (14.4%)	0.244
**New York heart association class, *n* (%)**			0.441
I	72 (23.0)	22 (17.6)	
II	186 (59.4)	78 (62.4)	
III	55 (17.6)	25 (20.0)	
IV	0 (0.0)	0 (0.0)	
**Deficient rims, *n* (%)**			
Upper	35 (11.2)	12 (9.6)	0.733
Atrioventricular valve	18 (5.8)	5 (4.0)	0.636
Aortic	92 (29.4)	38 (30.4)	0.908
Posterior	45 (14.4)	13 (10.4)	0.349
**Union score**	15.8 ± 2.1	15.6 ± 2.6	0.402
**Floppy septum, *n* (%)**	96 (30.7)	44 (35.2)	0.366
**Large size defects (≥25 mm), *n* (%)**	52 (16.6)	19 (23.2)	0.133
**Maximum size of defects, mm**	17.0 ± 8.9	17.3 ± 7.1	0.737
**Q_p_/Q_s_, ratio**	2.3 ± 0.5	2.2 ± 0.7	0.095

Values are mean ± SD or *n* (%); Qp/Qs: pulmonary/systemic flow ratio.

**Table 3:** Outcomes ^a^.

Category	Group T (*n* = 313)	Group E (*n* = 125)	Hazard Ratio (95% CI)	*p*-Value
**In-hospital outcomes**				
Procedure time, min	29.8 ± 15.3	41.5 ± 20.4	N/A	<0.001
Device size, mm	22.2 ± 3.3	22.4 ± 3.9	N/A	0.587
General anesthesia, *n* (%)	0 (0.0)	125 (100.0)	N/A	N/A
Succeeded at the first attempt, *n* (%)	275 (87.9)	108 (86.4)	0.878 (0.475–1.622)	0.750
Device size changing, *n* (%)	14 (4.4)	9 (2.9)	0.649 (0.277–1.523)	0.397
Conversion to open-chest surgery, *n* (%)	2 (0.6)	3 (2.4)	3.824 (0.631–23.165)	0.143
Transfusion, *n* (%)	1 (0.3)	0 (0.0)	0.997 (0.991–1.003)	>0.999
Length of stay in hospital, d	2.1 ± 2.3	2.9 ± 2.6	N/A	0.002
Shunt inside the device, *n* (%)	11 (3.5)	3 (2.4)	0.675 (0.185–2.462)	0.766
Device embolization, *n* (%)	1 (0.3)	1 (0.8)	2.516 (0.156–40.542)	0.490
Hemolysis, *n* (%)	1 (0.3)	0 (0.0)	0.997 (0.991–1.003)	>0.999
Device-related valve regurgitation, *n* (%)	0 (0.0)	1 (0.8)	1.008 (0.992–1.024)	0.285
Residual shunt, *n* (%)	1 (0.3)	0 (0.0)	0.997 (0.991–1.003)	>0.999
Atrial arrhythmias, *n* (%)	2 (0.6)	1 (0.8)	1.254 (0.113–13.955)	>0.999
Third-degree atrioventricular block, *n* (%)	0 (0.0)	1 (0.8)	1.008 (0.992–1.024)	0.285
Cardiac rupture, *n* (%)	1 (0.3)	1 (0.8)	2.516 (0.156–40.542)	0.490
Heart failure, *n* (%)	0 (0.0)	1 (0.8)	1.008 (0.992–1.024)	0.285
Death, *n* (%)	0 (0.0)	0 (0.0)	N/A	N/A
Hospital cost, USD ^b^	6233.3 ± 312.4	6673.7 ± 446.9	N/A	<0.001
**Long-term outcomes**				
Follow-up period, months	140.5 ± 33.8	138.8 ± 37.9	N/A	0.651
Device erosions, *n* (%)	1 (0.3)	1 (0.8)	2.516 (0.156–40.542)	0.490
Residual shunting, *n* (%)	0 (0.0)	0 (0.0)	N/A	N/A
Thrombosis events, *n* (%)	1 (0.3)	1 (0.8)	2.516 (0.156–40.542)	0.490
Heart failure, *n* (%)	2 (0.6)	1 (0.8)	1.254 (0.113–13.955)	>0.999
Atrial arrhythmias, *n* (%)	15 (4.8)	7 (5.6)	1.179 (0.469–2.964)	0.809
Third-degree atrioventricular block, *n* (%)	2 (0.6)	3 (2.4)	3.824 (0.631–23.165)	0.143
Stroke, *n* (%)	1 (0.3)	0 (0.0)	0.997 (0.991–1.003)	>0.999
Valve regurgitation, *n* (%)	2 (0.6)	1 (0.8)	1.254 (0.113–13.955)	>0.999
Death, *n* (%)	0 (0.0)	0 (0.0)	N/A	N/A
Total, *n* (%)	24 (7.7)	14 (11.2)	1.519 (0.758–3.042)	0.260

Values are mean ± SD or n (%); ^a^ All events were new during onset or had obviously deteriorated compared with the pre-procedural status. ^b^ Exchange rate was USD 1 for CNY 6.8.

## Data Availability

The data underlying this article cannot be shared publicly due to the privacy of the individuals that participated in the study. The data will be shared upon reasonable request to the corresponding author.
